# ApoE and ApoE Nascent-Like HDL Particles at Model Cellular Membranes: Effect of Protein Isoform and Membrane Composition

**DOI:** 10.3389/fchem.2021.630152

**Published:** 2021-04-29

**Authors:** Sarah Waldie, Federica Sebastiani, Martine Moulin, Rita Del Giudice, Nicolò Paracini, Felix Roosen-Runge, Yuri Gerelli, Sylvain Prevost, John C. Voss, Tamim A. Darwish, Nageshwar Yepuri, Harald Pichler, Selma Maric, V. Trevor Forsyth, Michael Haertlein, Marité Cárdenas

**Affiliations:** ^1^Department of Biomedical Science and Biofilms—Research Center for Biointerfaces, Malmö University, Malmö, Sweden; ^2^Institut Laue-Langevin, Grenoble, France; ^3^Partnership for Structural Biology (PSB), Grenoble, France; ^4^Department of Life and Environmental Sciences, Marche Polytechnic University, Ancona, Italy; ^5^Department of Biochemistry and Molecular Medicine, University of California, Davis, Davis, CA, United States; ^6^National Deuteration Facility, Australian Nuclear Science and Technology Organisation, Lucas Heights, NSW, Australia; ^7^Austrian Centre of Industrial Biotechnology, Graz, Austria; ^8^Graz University of Technology, Institute of Molecular Biotechnology, NAWI Graz, BioTechMed Graz, Graz, Austria; ^9^MAX IV Laboratory, Lund, Sweden; ^10^Faculty of Natural Sciences, Keele University, Staffordshire, United Kingdom

**Keywords:** ApoE isoforms, lipid exchange, reconstituted HDL, model membranes, neutron reflection, small-angle neutron scattering

## Abstract

Apolipoprotein E (ApoE), an important mediator of lipid transportation in plasma and the nervous system, plays a large role in diseases such as atherosclerosis and Alzheimer's. The major allele variants ApoE3 and ApoE4 differ only by one amino acid. However, this difference has major consequences for the physiological behaviour of each variant. In this paper, we follow (i) the initial interaction of lipid-free ApoE variants with model membranes as a function of lipid saturation, (ii) the formation of reconstituted High-Density Lipoprotein-like particles (rHDL) and their structural characterisation, and (iii) the rHDL ability to exchange lipids with model membranes made of saturated lipids in the presence and absence of cholesterol [1,2-dimyristoyl-sn-glycero-3-phosphocholine (DMPC) or 1-palmitoyl-2-oleoyl-glycero-3-phosphocholine (POPC) with and without 20 mol% cholesterol]. Our neutron reflection results demonstrate that the protein variants interact differently with the model membranes, adopting different protein conformations. Moreover, the ApoE3 structure at the model membrane is sensitive to the level of lipid unsaturation. Small-angle neutron scattering shows that the ApoE containing lipid particles form elliptical disc-like structures, similar in shape but larger than nascent or discoidal HDL based on Apolipoprotein A1 (ApoA1). Neutron reflection shows that ApoE-rHDL do not remove cholesterol but rather exchange saturated lipids, as occurs in the brain. In contrast, ApoA1-containing particles remove and exchange lipids to a greater extent as occurs elsewhere in the body.

## Introduction

Disorders in lipid metabolism are related to a range of diseases, among them atherosclerosis and Alzheimer's disease (AD). In atherosclerosis, the leading cause of death in western society, elevated levels of high-density lipoproteins (HDL) are thought to have counter-atherosclerotic properties (Gordon et al., 1989). However, the presence of HDL has more recently been shown to play a neutral (Voight et al., 2012) or even negative (Madsen et al., 2017) role in aiding the prevention of this disease. Even though HDL has been used to help combat the onset of atherosclerosis (*via* HDL therapy or plaque remodelling therapy) (Gille et al., 2014; Van Capelleveen et al., 2014), some recent medical trials have failed to prove HDL's effectiveness against atherosclerosis (Angeloni et al., 2013; Toth et al., 2013).

In general, HDL consist of a core of triglycerides and cholesterol esters, encased in a monolayer of lipids and cholesterol, surrounded by protein. There are many different subclasses of HDL each with slightly varying size and composition (Jonas, 2002). In particular, the lipid-poor, nascent HDL (also known as Preβ-HDL) is thought to be discoidal in shape and transforms into a spherical, mature HDL particle upon esterification of cholesterol and transfer into its lipid core. While ApolipoproteinA1 (ApoA1) constitutes around 70% of total protein content in human HDL, there are various other proteins which are also key to the structure and function of HDL particles (Jonas, 2002). One of these is ApolipoproteinE (ApoE) (Utermann, 1975) which is found in most HDL subfractions but mainly in the largest, most buoyant mature subfraction (HDL2) that contains triglycerides and are lipid rich (Davidson et al., 2009). ApoE is also the most commonly found apolipoprotein in cerebrospinal fluid (Ladu et al., 2009). It plays a large role in cholesterol transport while maintaining local homeostasis of cholesterol within the brain (Mahley, 2016b).

In humans, ApoE allelic variants result in three isoforms E2, E3, and E4, with respective frequencies of 8.4, 77.9, and 13.7% and varying at only two amino acid residues, 112 and 158 (E2: 112Cys, 158Cys; E3: 112Cys, 158Arg; E4: 112Arg, 158Arg) (Weisgraber et al., 1981). These differences in the amino acid sequence largely contribute to the proteins' structure and function and determine their behaviour in the body (Chou et al., 2005; Mahley et al., 2009), and especially in the roles they play in certain diseases such as atherosclerosis and AD. The presence of ApoE2 is associated with very low risk of AD (Wu and Zhao, 2016; Reiman et al., 2020) and generally thought to be protective against atherosclerosis, apart from in the case of type III hyperlipoproteinemia which is associated with increased atherosclerotic risk. On the other hand, ApoE3 levels are neutral in both diseases while the presence of ApoE4 is indicative of both atherosclerotic advancement and the onset of AD (Mahley et al., 2009).

Lipids are important to these diseases as they coexist in both atherosclerotic and AD plaques (Kiskis et al., 2015; Sergin et al., 2016). Understanding the difference in the interaction of ApoE variants with model cellular membranes is key to deciphering the specific roles they play in the development of these diseases. The roles of lipoproteins in the development of atherosclerosis and, most likely in AD, directly involve lipid exchange. The type of lipid exchange occurring— namely deposition or removal—highly depends on the lipoprotein type present (Browning et al., 2017). Previously, HDL was shown to remove lipids from the cell membrane giving rise to the idea behind HDL therapy. Reconstituted HDL (rHDL), comparable to discoidal Preβ-HDL, exhibits similar lipid transfer properties to that of mature HDL (Davidson et al., 1995), and is used to mimic this, discoidal nanoparticles of phospholipids solubilised by encircling ApoA1 are used as artificial HDL or rHDL as a strategy in HDL therapy (Gille et al., 2014; Van Capelleveen et al., 2014). While less is known about the lipid exchange properties of ApoE, ApoE-containing rHDL (ApoE-rHDL) were shown to have potential for atherosclerosis treatment (Valanti et al., 2018), although further testing is needed.

In this paper, the initial interaction of ApoE3 and ApoE4 with saturated and unsaturated supported lipid bilayers (SLBs) is explored using neutron reflectometry (NR). Then, ApoE-rHDL are formed based on the vesicle solubilisation method often used to make ApoA1-rHDL. The ApoE-rHDL structure is further characterised using small angle neutron scattering (SANS). Finally, the lipid exchange capacity for ApoE-rHDL with model membranes is explored by NR. As ApoE is the main apolipoprotein in the brain, where there is a local homeostasis of cholesterol, the model membranes in question are in the absence or presence of cholesterol to determine the potential differences between these interactions. Comparisons are made between to ApoA1-based rHDL and mature HDL pooled from three healthy male volunteers [mature HDL is replotted from Waldie et al. (2020)].

## Materials and Methods

### Materials

MilliQ water (18.2 MΩ cm) was used for all experiments, solvent preparations and cleaning procedures. D_2_O (99.9% deuterated, Sigma-Aldrich) was provided by the Institut Laue-Langevin (ILL), Grenoble, France. Bradford reagent, calcium chloride (CaCl_2_) and Tris buffer saline (TBS) tablets were from Sigma-Aldrich. Tris buffer (50 mM Tris, 150 mM NaCl, pH 7.4: TBS) was prepared by dissolving a tablet in H_2_O as specified by the producer or D_2_O to the same concentration. 1,2-dimyristoyl-sn-glycero-3-phosphocholine (>99%, hDMPC), 1,2-dimyristoyl-d54-sn-glycero-3-phosphocholine (>99%, dDMPC) and cholesterol (h-cholesterol; >98%) were from Avanti Polar Lipids (Alabaster, AL). Tail deuterated 1-palmitoyl, 2-oleoyl-sn-glycero-3-phosphocholine (d67; overall tail deuteration 95%, dPOPC) was provided by the deuteration facility at ANSTO, produced and purified as previously described (Yepuri et al., 2016).

### Protein Expression and Purification

BL21DE3 *Escherichia coli* cells were transformed with pET-32a(+) plasmids containing either ApoE3 or ApoE4 and ampicillin resistance. Cells were cultivated in Terrific Broth medium (12 g L^–1^ tryptone, 24 g L^–1^ yeast extract, 4 mL L^–1^ glycerol, 9.4 g L^–1^ dipotassium phosphate and 2.2 g L^–1^ potassium dihydrogen phosphate) at 37°C. When cultures reached an OD_600_ nm of 0.6–0.8, protein expression was induced by adding 1 mM isopropyl-beta-thiogalactopyranoside (IPTG) in the culture medium and incubated for a further 90 min. The cells were then harvested by centrifugation (19,000 rpm, JLA 9.1000 rotor 20 min, 4°C) after which the cell pellets were resuspended in TBS buffer and sonicated on ice for 10 min (50% power, 30 s on, 30 s off). hDMPC was suspended in water by bath sonication for 30 min and then added to the protein at a concentration of 100 mg per 1 L of culture and dialysed against TBS at room temperature overnight. The hDMPC is added to protect the hinge region of ApoE when cleaving with thrombin. After dialysis, thrombin was added in excess for 6 h at 37°C. The sample was analysed by SDS-PAGE to ensure all fusion protein had been cleaved. Potassium bromide was added to the ApoE-DMPC mixture to a density of ~1.21 g mL^–1^. Ultra-clear tubes (Beckman) were filled two-thirds with a ~1.12 g mL^–1^ density solution (16% w/v potassium bromide solution dissolved in 20 mM Tris pH 7.4 and 0.05% w/v sodium azide) and underlayed with the lysate solution. Samples were spun for 16 h at 38,000 rpm in a SW41 rotor (Beckman) at 15°C with the break off to preserve the gradient. The resulting “floating pellet” was recovered and dialysed against TBS to remove the potassium bromide salts. The ApoE-DMPC complexes were lyophilised and frozen at −20°C. When fresh protein was required the pellets were delipidated against methanol and resuspended in 6 M Guanidine Hydrochloride (GuHCl) 50 mM Tris pH 8 and 0.5% beta-mercaptoethanol (BME), dialysed against 4 M GuHCl, 10 mM Tris pH 7.5, 1 mM EDTA, and 0.1% BME; further against 100 mM ammonium bicarbonate; and finally into TBS. The protein was then purified *via* gel-permeation column chromatography on 2 S200 columns in series and ready to use.

### Protein-Lipid Particle Production and Purification

To form the ApoE-rHDL particles, equal volumes of fresh protein are mixed with freshly extruded hDMPC vesicles at a final molar ratio of 1:100 ApoE:DMPC. The hDMPC vesicles were produced by first forming a thin lipid film (in small vials, under nitrogen flow under manual rotation) that was suspended in TBS first by vortexing, then by bath sonication for 1 h. The lipid suspension was extruded 41 times using an Avanti extruder and a filter of 100 nm pore size (Millipore). The solution was incubated at 24°C for 12 h or overnight. Verification of the particle formation was carried out *via* dynamic light scattering due to a slight reduction in the peak intensity for the 100 nm vesicles and the presence of a small peak at roughly 10–15 nm. The rHDL particles were purified *via* gel filtration chromatography using a Superose 6 10/300 column.

### Deuterated Cholesterol Production

The production of tailor-deuterated cholesterol made use of the Deuteration Laboratory within the Life Sciences Group (Haertlein et al., 2016) at the ILL. Based on previous developments for the production of perdeuterated cholesterol (Moulin et al., 2018), matchout-deuterated cholesterol (d-cholesterol) was produced and purified as reported previously (Waldie et al., 2019). The Pichia pastoris strain CBS7435 Δhis4Δku70 Δerg5::pPpGAP-ZeocinTM-[DHCR7] Δerg6::pGAP-G418[DHCR24] was grown in 100% deuterated basal salts medium in the presence of non-deuterated glycerol as the sole carbon source. The batch phase was complete after 7 days in a fermenter at 28°C; the fed-batch phase was initiated by constant feeding of glycerol for a further 12 days. The cells were harvested and then isolated using an organic solvent extraction method followed by HPLC to obtain pure cholesterol, verified by GCMS.

### Model Membrane Preparation

Lipid films were prepared in small glass vials from chloroform stocks of dDMPC, dPOPC, h- and d-cholesterol. Twenty mol% cholesterol was incorporated into the cholesterol-containing films. The films were dried under a stream of nitrogen under manual rotation, and placed under vacuum overnight. Before use, the lipid films were hydrated in MilliQ water, vortexed and bath sonicated for 1 h. Immediately prior to injection the lipids were tip sonicated for 5 min (20% power, 5 s on, 5 s off), mixed with an equal volume of 4 mM CaCl_2_ and injected into the pre-equilibrated NR solid-liquid flow cells by a syringe port (Browning et al., 2017; Waldie et al., 2019). The presence of 2 mM CaCl_2_ and concentration of 0.1 mg mL^–1^ of lipids were used to optimise vesicle fusion (Åkesson et al., 2012a; Waldie et al., 2018). The lipids were incubated for 20 min before rinsing with water, followed by 50 mM Tris saline buffer, pH 7.4. This process leads to a supported lipid bilayer or “model membrane.”

### Scattering

SANS and NR data were collected; scattered intensity and reflectivity, respectively, were measured as a function of momentum transfer, *q* = *4*π*sin(*θ*)/*λ, where θ is half the scattering angle for SANS and the incident angle for NR and λ is the neutron radiation wavelength for both.

#### Neutron Reflectometry

Neutron reflectometry data were collected on the time-of-flight reflectometer FIGARO (Campbell et al., 2011, 2015) at the ILL (Grenoble, France). Momentum transfer ranges of 0.01 > q > 0.3 Å^−1^ were measured using wavelengths 2 < λ < 20 Å and two incident angles, 0.8 and 2.3°, with a spatial resolution (Δq/q) of 7%. The area exposed to the neutron beam was 30 × 60 mm^2^. The experiments were carried out in reflection-up mode to ensure no aggregated particles could settle on the surface being measured. The analysis of specular reflectivity data allowed a scattering length density (SLD) profile perpendicular to the surface, to be obtained.

The silicon (111) blocks were treated with Piranha solution (H_2_SO_4_:H_2_O_2_, 7:3) for 10 min at 80°C before extensive rinsing with MilliQ water (Warning: Piranha solution reacts violently with organic materials and should be handled with extreme caution). The polyether ether ketone (PEEK) and O-ring components of the cells were thoroughly cleaned in Hellmanex 2% (v/v) solution and MilliQ water twice *via* bath sonication, with rinsing of MilliQ water between each sonication. Solvent contrasts were changed *in situ via* HPLC pump. Three isotopic contrasts were used: 100% h-TBS (TBS made using H_2_O), 100% d-TBS (TBS made using D_2_O) and 38% d-TBS (or cmSi) to contrast-match the silicon block.

The MOTOFIT programme (Nelson, 2006) was used to simultaneously fit the three isotopic contrasts for each experimental data set, and the Monte Carlo error analysis using genetic optimisation within the software was used to determine the error of the fits. The significance of pairwise parameters were calculated using an *F*-test assuming normal distributed errors. The clean silicon surfaces were characterised initially to determine the thickness and roughness of the oxide layer. After bilayer deposition, data was fitted using a symmetrical leaflet model (heads-tails-heads) using either a four- or five-layer model for the membranes since some samples required an extra solvent layer between the silicon oxide and the bilayer. Symmetry implies that the thickness, coverage and SLD of both headgroup layers are the same, and the roughness was constrained across all regions of the bilayer. The models were constrained to have the same mean molecular area (MMA), within error, across the different lipid bilayer layers. This fitting approach gives equivalent results to the molecularly constrained model (for which MMA is used as a fitting parameter instead). For example, POPC bilayers measured at 25°C were reported to be 62 Å^2^ (Luchini et al., 2019) and 62 Å^2^ (Åkesson et al., 2012a) fitting individual thicknesses or MMA, respectively.

After bilayer characterisation, the ApoE protein or protein-lipid particles were introduced into the solid-liquid flow cells *via* syringe pump at a rate of 1 mL min^−1^. The protein and rHDL concentrations were 0.075 mg mL^−1^ and 0.132 mg mL^−1^, respectively. After either 6 or 8 h of incubation, the bilayers were rinsed with buffer and re-characterised in all buffer contrasts. When fitting the bilayers post-interaction, the initial parameters were used as a starting point (Table 1) while keeping constant the silicon oxide layer parameters. For some bilayers, acceptable fits were obtained by varying only the SLB coverage and lipid tail SLD. For other bilayers, this approach gave no suitable fits forcing the use of the thickness of these regions as an additional fitting parameter. Moreover, an extra layer on top of the bilayer was necessary in all cases corresponding to either the protein or rHDL particles still attached to the bilayer.

**Table 1:** Representative structural parameters for the SLB used.

	**Head group thickness/**	**Tail thickness/**	**Headgroup coverage / %**	**Tail coverage / %**	**Mean molecular area / Å^**2**^**
	**Å**	**Å**			
dDMPC	9.0 ± 0.1	27.2 ± 0.2	62 ± 1	96 ± 1	59 ± 2
dPOPC	8.8 ± 0.2	28.6 ± 0.4	54 ± 2	86 ± 1	71 ± 5
dDMPC d-cholesterol*	8.5 ± 0.2	32.1 ± 0.2	71 ± 1	98 ± 1	52 ± 4
dDMPC h-cholesterol*	7.7 ± 0.4	30.7 ± 0.5	85 ± 5	94 ± 1	51 ± 4

*Corresponding NR profiles and best fits are given in the Supplementary Information*.

* *The model membranes containing cholesterol had a nominal composition of 80:20 mol %, and its real cholesterol content was determined to be the nominal one within error (Waldie et al., 2018). d- and h- cholesterol refer to deuterated and hydrogenated cholesterol respectively*.

#### Small-Angle Neutron Scattering

SANS data were collected on the D11 instrument at the ILL (Lindner and Schweins, 2010). The experiments were carried out at 25°C with a momentum transfer range of 0.002 < q < 0.3 Å^−1^ by using detector distances of 1.4, 8, and 39 m at constant wavelength λ = 6 Å (fwhm 9%).

The protein-lipid particles were measured in three contrasts: 100% h-TBS, 100% d-TBS and 42% d-TBS to contrast-match the protein. The data were corrected for the empty cell, background and the absolute scale was obtained from the attenuated direct beam measurement and validated from the level of water (1 mm, H_2_O), used as a secondary standard. The cells were 1 mm path-length QS quartz glass Hellma SANS cuvettes (Hellma GmbH, Müllheim, Germany) and the data reduction was carried out using LAMP. The SasView programme was used to fit the experimental data^1^. The three contrasts were fitted simultaneously to constrain the fit.

## Results

**Figure 1:**
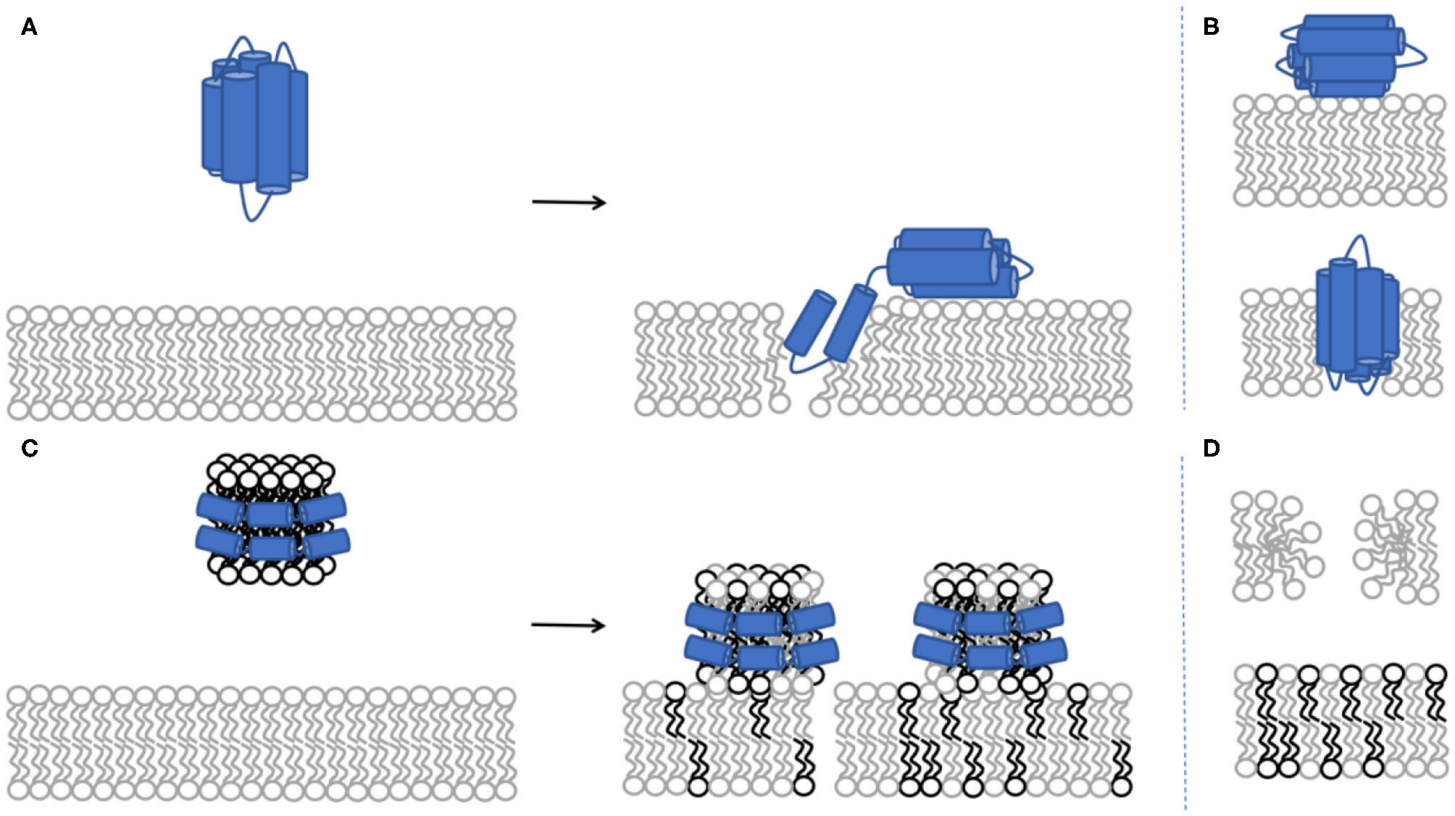
Schematic representation of protein incorporation into the phospholipid bilayer **(A)**. NR cannot distinguish between the conformation of the protein upon binding to the model membrane: whether there are two individual protein molecules adsorbing either in the core or the headgroup region vs. a single individual protein molecule bending across the membrane **(B)**. Schematic representation for lipid exchange between the rHDL and the phospholipid bilayer **(C)**. Lipid exchange represents the combination of lipid removal from the model membrane and lipid deposited by the rHDL particles **(D)**. The light grey colour represents deuterated lipids while the black colour represents non-deuterated lipids. Both DMPC and POPC lipids were used above their melting temperature, giving fluid supported lipid bilayers. Addition of 20 mol% cholesterol to either POPC or DMPC give SLBs with fluid properties (Waldie et al., 2020).

In this work, the overall journey of ApoE is followed from its interactions with model membranes in the lipid-poor form, to the formation of a nascent-like HDL particle, and finally to the characterisation of the lipid exchange capacities for saturated lipids in the presence or absence of 20 mol% cholesterol. NR and SANS are ideal techniques for this task as they can distinguish between deuterated and non-deuterated components. Therefore, the use of deuteration in lipid-protein complexes allows highlighting of specific components within the system, together with the use of contrast variation (based on the different content of D_2_O-based buffers, denoted as d-TBS) to visualise the differences between the components (Clifton et al., 2020). Here, the model cellular membranes are made up of tail-deuterated lipids and, in some membranes, the presence of non-deuterated or specifically matchout-deuterated cholesterol. The matchout deuterated cholesterol allows no net coherent scattering to be seen for this component in 100% D_2_O-based buffer conditions. The protein and rHDL particles, instead, are unlabelled. The differences between the components in the membrane and the protein/rHDL allows NR to be used in determining: (1) the level of protein incorporation or lipid exchange, through the change in the scattering length density (SLD) of the tail region, (2) the amount of lipids that were removed and replaced by protein or solvent, through the change in the solvent quantity in the tail region, and (3) the amount of extra protein/rHDL bound on top of the model membrane. During the kinetics of interaction, the samples were measured in H_2_O-based buffer (100% h-TBS) as this gives the best contrast against the deuterated membrane layer, while full characterisation in three contrasts (100% h-TBS, 100% d-TBS and 38% d-TBS) of the model membranes before and after kinetics were measured to increase the sensitivity and accuracy of the structural and compositional information obtained. The layer models describing the model membranes are determined from simultaneous fitting of the isotopic contrasts and allow for the analysis of the membrane composition and the decoupling of the information regarding protein incorporation/lipid exchange and lipid removal as described in the methods section and the Supporting Information. Figure 1 gives schematic representations of protein and nanodisc interactions with the bilayers. Representative structural parameters for the pristine SLBs are summarised in Table 1 and are in agreement with those reported in the literature for DMPC (Browning et al., 2018), POPC (Åkesson et al., 2012b; Waldie et al., 2020), 20 mol% cholesterol containing DMPC and POPC measured at 37°C (MMA increases with temperature and the reported values cannot be compared to MMA measured at 25°C (Åkesson et al., 2012a). POPC is fond to adopt a thicker, more expanded SLB structure than DMPC given its longer tail and the presence of double bonds that bend the acyl tails. Here, the thickening and compacting effect of cholesterol on fluid membranes is observed in agreement with previous reports (Gallová et al., 2004; Hung et al., 2007; Waldie et al., 2018, 2019). Moreover, the cholesterol molecules are expected to be fully miscible within these model membranes (Knoll et al., 1985; Barrett et al., 2013).

The samples measured by SANS (all unlabelled) were measured in three contrasts: 100% h-TBS, 100% d-TBS and 42% d-TBS. The different buffers used give varying levels of isotopic contrast; for example, the 42% d-TBS and 100% h-TBS highlight the lipids and the proteins respectively, whereas the 100% d-TBS buffer gives the largest contrast against the sample as a whole. Analysing all three contrasts simultaneously allows for higher sensitivity in the structural determination.

### Initial Apolipoprotein Interaction With Model Membranes

**Figure 2:**
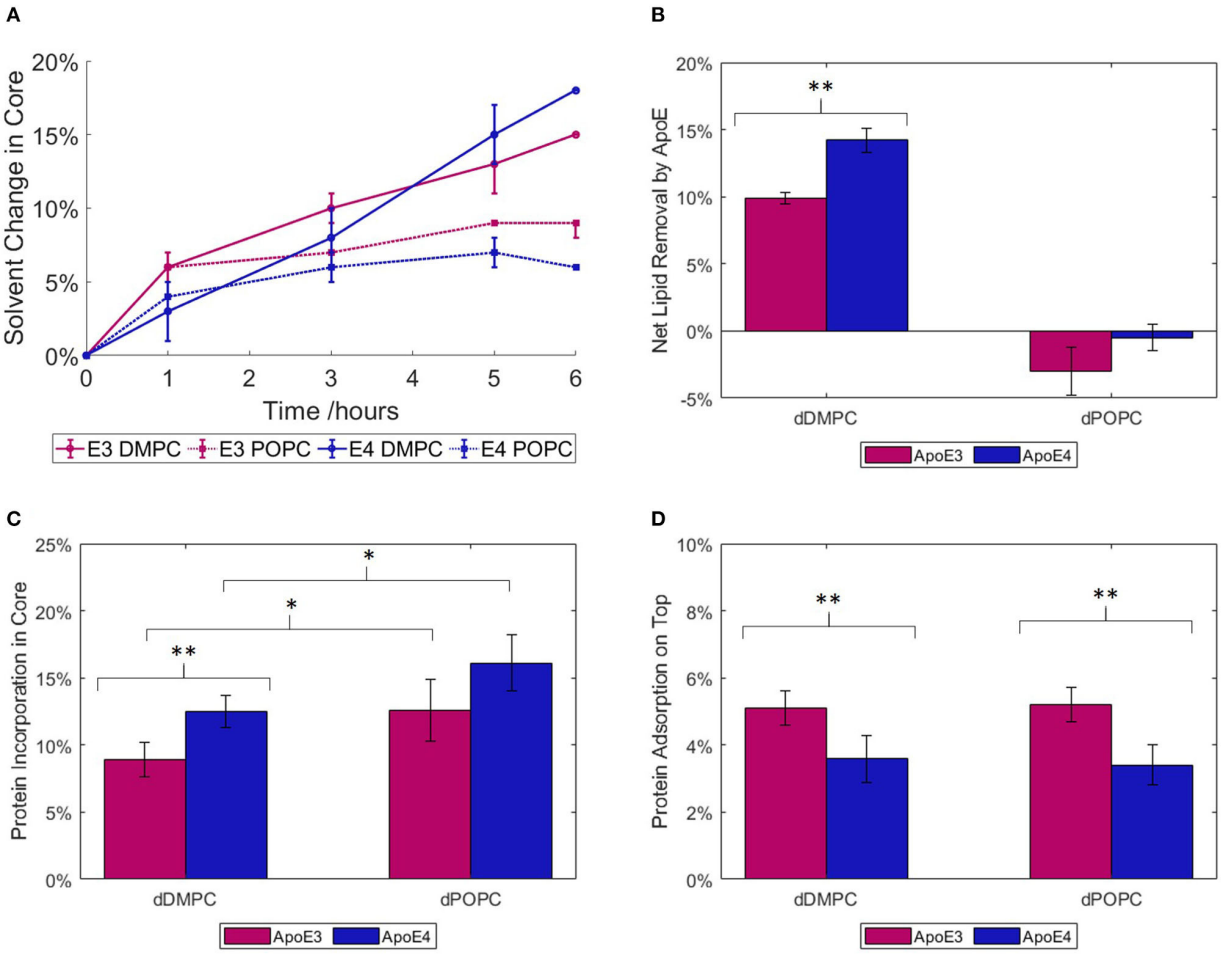
ApoE interaction with saturated (dDMPC) or unsaturated (dPOPC) model membranes measured at 37°C, in Tris-buffer at pH 7.4: kinetics of lipid replacement in terms of the relevant change in solvent penetration of the lipid core, taking into account SLD change also, are given for ApoE3 and ApoE4 **(A)**. Net lipid removal calculated as the difference in solvent coverage within the bilayers before and after 6 h incubation with apolipoproteins **(B)** and volume fractions of protein binding within the core **(C)** or on top of **(D)** the SLBs upon 6 h of incubation and rinsing with Tris buffer. The NR profiles and best fits are shown in the Supplementary Figures 1, 2. *Statistically different assuming *p* = 0.1; **Statistically different assuming *p* = 0.05.

The binding of ApoE3 or ApoE4 to model membranes comprised of saturated or unsaturated phospholipids was followed by NR as a function of the saturation level in the bilayer at 37°C in Tris buffer, pH 7.4 (Figure 2). For this, we used saturated, tail deuterated dDMPC and unsaturated, tail deuterated dPOPC. As shown in Figure 2A, in the case of the saturated lipids (dDMPC), ApoE4 presents a slightly stronger interaction with the lipids than ApoE3 in terms of lipid removal and lipid replacement by protein adsorption (reflected by the solvent change in the membrane core), whereas the opposite trend can be observed for the unsaturated lipids (dPOPC). In terms of kinetics, most of the binding to unsaturated membranes occurs within the first hour of incubation for both ApoE isoforms (Figure 2A). For saturated lipids, the two ApoE variants show a slightly different behaviour. On one hand, ApoE3 shows a similar initial pattern to that of unsaturated lipids but this is followed by a slower increase, possibly suggesting a gradual and slower removal of saturated lipids over longer time periods. On the other hand, ApoE4 follows a more linear increase with time. This implies a difference in the mode of interaction of the proteins with the model membranes and this interaction seems to be variant-dependent and sensitive to the level of lipid saturation.

Following the incubation period, NR data were collected using three isotopic contrasts to enable quantification of lipid removal (calculated from change in solvent penetration), protein binding to the membrane hydrophobic core and adsorption on top of the membrane (Figures 2B–D). This additional 28 Å layer represents most of the compact protein in a lipid-free state, allowing for some part to rearrange and integrate itself into the bilayer (Chen et al., 2011). The main lipid-binding region is the C-terminal (Li et al., 2003), thus it is possible that the protein could rearrange itself to allow maximum binding of lipids to this area. For membranes incubated with ApoE3, few changes were required from the pristine membrane (lipid bilayer) structure: the lipid head and tail thicknesses remained constant, while there were minor changes in the lipid tail SLD and both head and tail solvation. On the other hand, interaction with ApoE4 required additional changes in the head and tail thicknesses to obtain better fits to the NR data. The increased membrane modifications seen with both isoforms correlate to the increased levels of embedded protein. When looking at the net lipid removal (or increase in solvation) and protein insertion (due to changes in SLD of the membrane hydrophobic core) upon incubation with ApoE, both variants showed a preference for the removal of saturated lipids rather than unsaturated ones (Figure 2B). Lipids were removed and replaced by protein insertion into the bilayer core (Figure 2C) with more protein incorporation into unsaturated than saturated membranes. The latter, in turn, indicates that the protein is more prone to remain bound to the unsaturated membrane without actually removing lipids. Additionally, a further protein layer is formed on top of the SLB (Figure 2D). This layer has a greater coverage, or protein volume fraction, for ApoE3 than for ApoE4 regardless of membrane type. This indicates that ApoE3 preferentially interacts with the lipid headgroups compared to ApoE4. Interestingly, and regardless of membrane type, the protein fraction that binds to the lipid bilayer core is larger for ApoE4 than ApoE3. Indeed, 70 and 76% of the ApoE4 protein co-localised within the core of the saturated and unsaturated membranes respectively, while these fractions decreased to 54 and 62% for ApoE3. NR can though not distinguish whether it is the same protein stretching across the core and the heads, or different individual proteins occupying either the head or tail region in the SLB. In summary, even though both ApoE variants displayed more binding to unsaturated bilayers, the NR data highlight a difference between the variants. In particular, the ApoE4 isoform binds to a larger extent to the lipid core than the ApoE3 isoform and the conformation of this fraction is less sensitive to the type of lipid in the model membrane.

### ApoE-Containing Nascent-Like HDL Particle Structure

**Figure 3:**
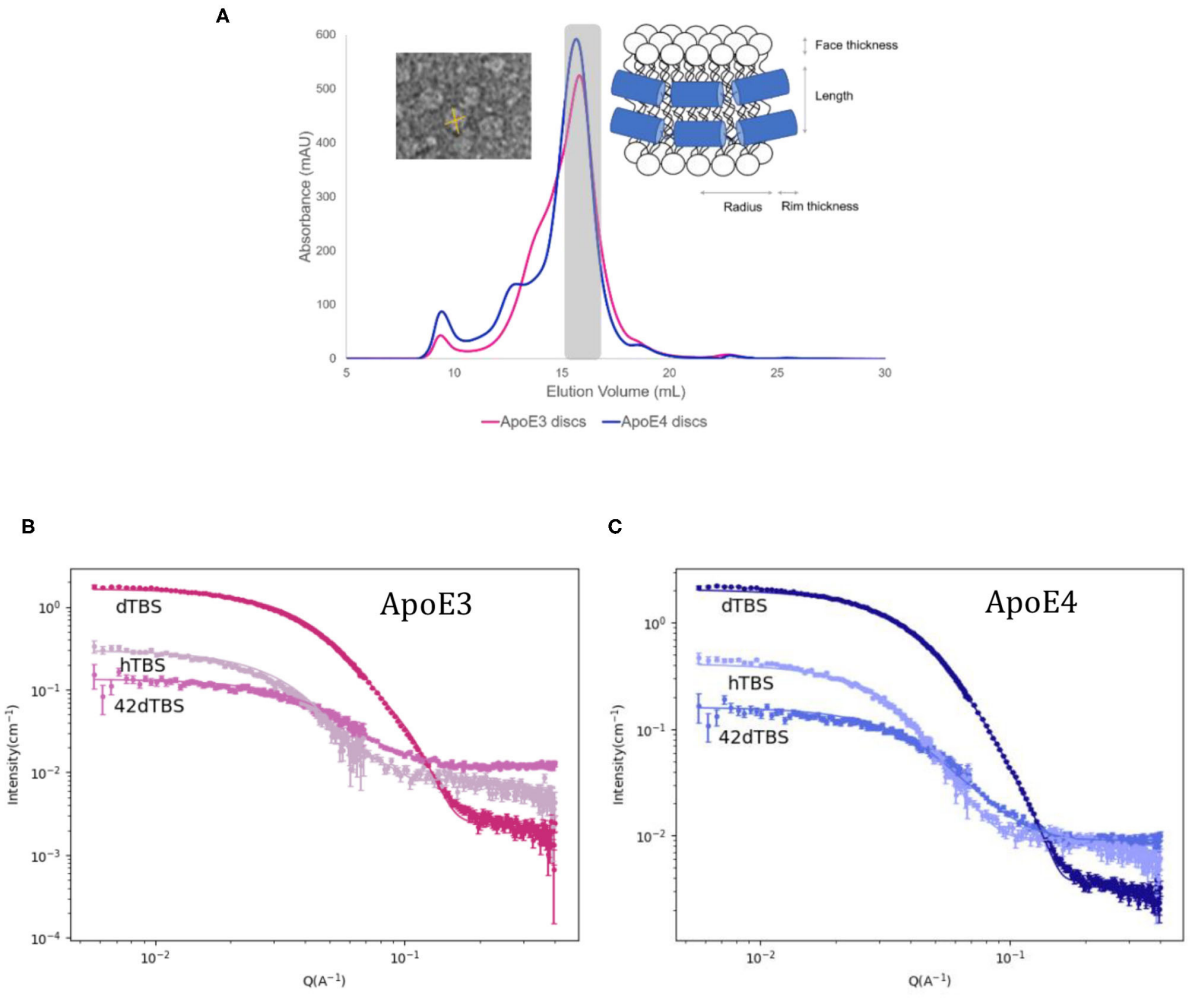
Size exclusion chromatograms for ApoE3 and ApoE4 discs with inset of model used for fitting and negative stained TEM images for ApoE3-rHDL **(A)** SANS data and best fits in three contrasts for the ApoE3- **(B)** and ApoE4-rHDL **(C)** measured at 25°C in Tris buffer pH 7.4. The parameters used for the best fits shown are listed in Table 2.

rHDL made of ApoE and hDMPC were prepared using the vesicle solubilisation approach and purified by size exclusion chromatography (Figure 3A). The resulting rHDL particles were structurally characterised by SANS in three isotopic contrasts to highlight different parts of the particle and to increase the accuracy of the fit (Figure 3B). For the fitting, the “nanodisc” like structure model was adopted, which consists of an elliptical bilayer encased by protein (inset of Figure 3A) and both fitted and calculated values can be found in Table 2. These discs resemble nascent HDL. HDL are mainly present in cerebrospinal fluid but are also important for the clearance of lipids in the liver *via* binding to ApoE receptors (Pitas et al., 1987).

**Table 2:** Fitted and calculated parameters for the protein-DMPC particles.

**Parameters**	**ApoE3**	**ApoE4**
Radius^*^ Å	42.0 ± 0.4	38.1 ± 0.2
Ellipticity ratio^*^	1.4	1.7
Protein rim thickness^*^ Å	11.0 ± 0.8	8.6 ± 0.3
Lipid headgroup thickness^*^ Å	9.0 ± 0.1	9.0 ± 0.1
Bilayer core thickness^***^ Å	28	28
Short-long axis disc diameter^**^ Å	106–139.6	93.4–146.7
Disc circumference^**^ Å	389	386
No. amino acids^**^	260	258
Area per lipid^**^ Å^2^	55.9	55.9
No. lipids per leaflet^**^	139	139
No. proteins per disc^**^	2	2

* *Fitted values*.

** *;Calculated values*.

*** *Fixed value*.

The calculated values from Table 2 are determined as follows. The equation used for circumference determination takes into account the ellipticity of the particles as used previously (Skar-Gislinge et al., 2010):

(1) $$\begin{array}{l} Circumference\;=\\ \;2\pi \sqrt{[{\left(r_{minor}+d_{belt}\right)}^{2}+\;{\left(r_{major}+d_{belt}\right)}^{2}]/2} \end{array}$$

where *r*_minor_, *d*_belt_ and *r*_major_ are the radius of the short axis, the width of the protein belt and the radius of the major axis respectively. This can be used for the determination of the number of amino acids in contact with the bilayer by using an average length per amino acid of 1.5 Å. With 299 residues per ApoE molecule, this gives a maximum circumference of ~ 448 Å which is slightly larger than what was calculated here, however leaves room for flexibility in the protein and possible expansion. Previously reported data on related particles prepared with similar proteins have also allowed for some residues to not be in contact with the bilayer: about 20 amino acids per protein were estimated not to be in direct contact with the lipid core (Denisov et al., 2004; Skar-Gislinge et al., 2010). The resulting area of the lipid bilayer region gives 139 lipid molecules per leaflet for both ApoE3 and ApoE4, and each disc contains two proteins, one per leaflet. This value is in agreement with the one measured by phosphate analysis (145 and 151 lipids per leaflet for ApoE3 and ApoE4, respectively). This number of lipids per leaflet agrees with data provided for similar length apolipoprotein-like proteins (Bayburt and Sligar, 2010). The mean molecular area for the heads and tails was calculated to be ~56 Å^2^ with ~30% hydration in the heads which agrees well with the NR data presented and values found in the literature (Bayburt and Sligar, 2010). The disc diameter is larger compared to ApoA1 discs prepared in a similar manner (Midtgaard et al., 2015); however, ApoE (34 kDa) is substantially longer than ApoA1 (28 kDa) (Lund-Katz and Phillips, 2010), thus resulting in larger discs. The diameter of the particles is in agreement with dynamic light scattering data measured for both ApoE3 and ApoE4 based discs which gave values between 10 and 15 nm prior to SANS measurements (results not shown). Both ApoE3 and ApoE4 gave similar structured discs with the same number of lipid molecules per leaflet and number of proteins. This is not surprising due to the same protein length and very similar sequence, only differing in one amino acid. The main structural difference in the discs found between the ApoE variants was the ellipticity, with ApoE3-rHDL being less elliptical than ApoE4-rHDL. Finally, allowing the bilayer core thickness to vary between a reasonable range did not significantly improve the fit quality, thus it was kept fixed at 28 Å as it was similar to the value seen with the NR. The use of atomic force microscopy can also be beneficial to measure such thicknesses (Bayburt and Sligar, 2002).

### ApoE-Containing rHDL at Model Membranes

**Figure 4:**
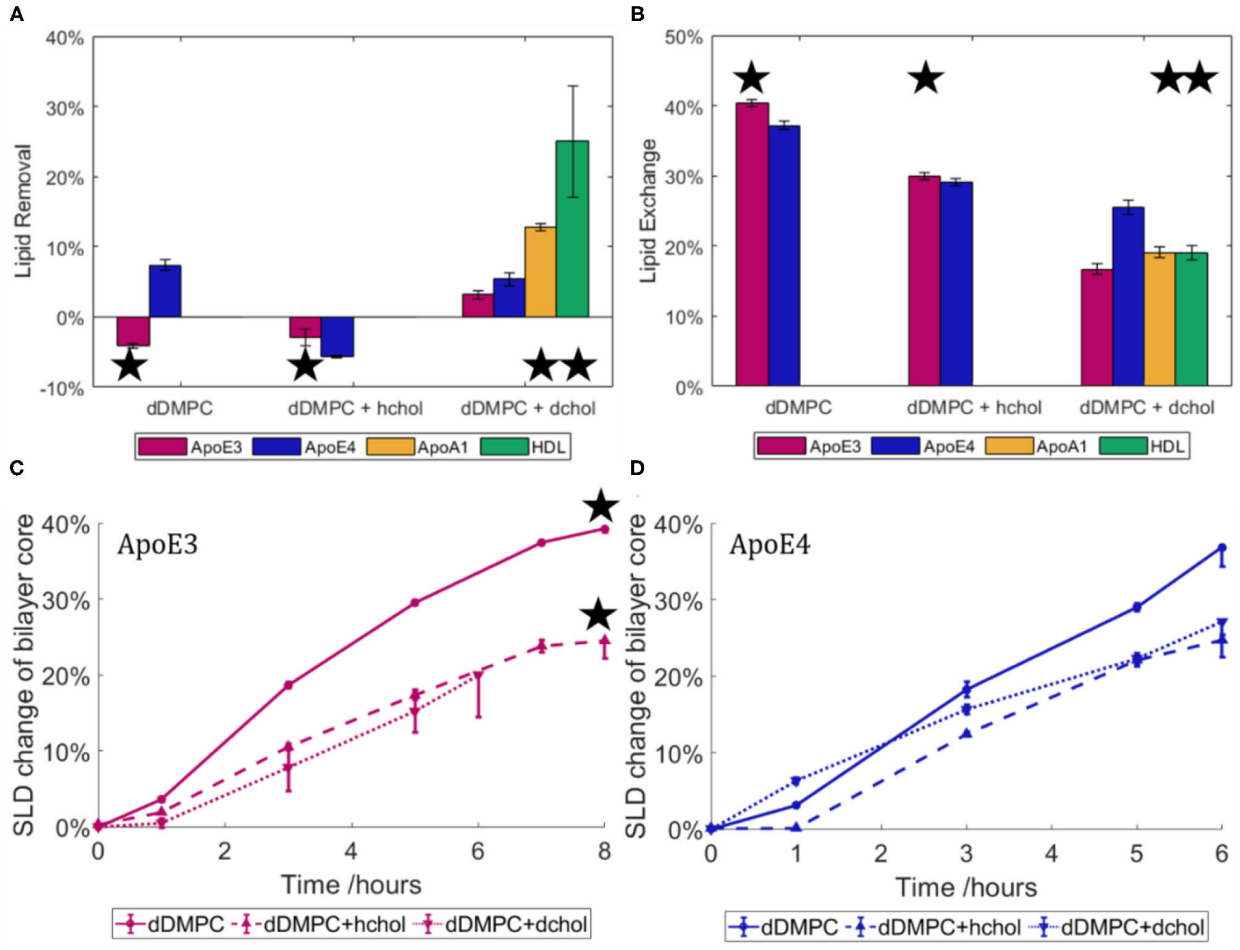
Lipid removal **(A)** and lipid exchange **(B)** across model membranes for ApoE3- and ApoE4-rHDL. Three model membranes were used: saturated lipids (dDMPC) in the absence or presence of hydrogenous or deuterated cholesterol. Data for mature HDL and ApoA1-rHDL against the cholesterol containing bilayers (Waldie et al., 2020) are also included. The asterisk indicates an incubation time of 8 h compared to 6 h for those without. Kinetics of lipid exchange for rHDL containing ApoE3 **(C)** and ApoE4 **(D)** in terms of the relative change in SLD of the solvated lipid core over time. HDL data is replotted from Haertlein et al. (2016). The NR profiles and best fits are given in the Supplementary Figures 4, 5. All statistically different assuming *p* = 0.05 apart from dDMPC + hchol for lipid exchange **(B)**.

As ApoE isoforms play different roles in the onset and development of atherosclerosis and AD, with lipids found in both atherosclerotic and AD plaques, we therefore used NR to determine how the nascent-like rHDL of each isoform behave in the exchange or removal of lipids. To this end, ApoE-rHDL particles were incubated with model membranes composed of dDMPC, in the absence or presence of cholesterol, at 37°C in Tris buffer at pH 7.4, and their interactions were followed by NR (Figure 4). Both hydrogenous (h-cholesterol) and deuterated cholesterol (d-cholesterol) were used to determine whether ApoE particles specifically targeted the cholesterol molecules.

As shown in Figure 4A, there was little change in the surface coverage of the model membrane upon interaction with ApoE-rHDL as all SLBs started with at least 95% coverage and finished with no less than 90% coverage. At similar protein concentration, such small capacity for lipid removal cannot be compared to mature HDL (purified from 3 male, healthy adult donors) (Waldie et al., 2020), which can remove about 40% of the model membrane (Waldie et al., 2020) in the absence of cholesterol. This shows that, while some lipids were removed in certain cases, lipid removal is not the main role of the ApoE nascent-like HDL. HDL particles contain a large number of apolipoproteins (Maric et al., 2019), with ApoA1 being the most abundant in serum. Thus, the interaction of ApoA1-rHDL (also prepared by the vesicle solubilisation method (Del Giudice et al., 2017a,b) with a cholesterol containing saturated membrane was also investigated (Del Giudice et al., 2017a,b). In the case of ApoA1-rHDL, significant lipid removal occurred, though to a lesser extent than mature HDL. Lipid removal was double for ApoA1-rHDL and as much as five times for mature HDL as compared to ApoE-rHDL samples. This suggests that more than just the increased incubation time (ApoA1-rHDL and HDL were incubated for 8 h rather than 6 h) is at play. Additionally, kinetics of lipid exchange show that equilibrium was reached after 6 h for ApoE3-rHDL (Figure 4C) and ApoA1 (Supplementary Figure 5) while continuous increase was observed for ApoE4-rHDL (Figure 4D), regardless of membrane composition.

Lipid exchange (Figure 4B) is hereby defined as lipids removed from the model membrane and replaced with lipids from the rHDL particles, and is calculated from the SLD change within the lipid tail region. In this case, a significant proportion of the lipids were exchanged by ApoE-rHDL and the extent of which was similar regardless of the ApoE isoform present. In the absence of cholesterol, 40.4 ± 0.5% and 37.2 ± 0.6% lipid exchange occurred for ApoE3- and ApoE4-rHDL respectively. However, in the presence of cholesterol, 30.0 ± 0.5% and 29.1 ± 0.5% lipid exchange took place for ApoE3- and ApoE4-rHDL respectively. Bearing in mind the difference in incubation time (ApoE3-rHDL were incubated 2 h longer than ApoE4-rHDL, see Figures 4C,D), this suggests that ApoE4-rHDL have the ability to exchange more lipids than ApoE3-rHDL. This is confirmed when observing the quantities of lipids exchanged in the membranes containing d-cholesterol for which the samples were incubated for the same time period (6 h). In this case, the final exchange values differ more drastically: 16.7 ± 0.8% and 25 ± 1% for ApoE3 and ApoE4, respectively. The trend is clear that ApoE4-rHDL exchanged more lipids than the ApoE3-rHDL regardless of membrane composition. In Figure 4B, the extent of lipid exchange for both ApoA1-rHDL and mature HDL upon 8 h incubation has similar lipid exchange capacity to ApoE3-rHDL, while ApoE4-rHDL has significantly greater affinity for lipid exchange than any of the other samples tested.

To be able to determine if cholesterol was being preferentially exchanged by either ApoE variant, both deuterated and non-deuterated cholesterol were used. If cholesterol was exchanged preferentially over the phospholipids, a net difference would be seen in the final quantity of lipids exchanged across these two membranes due to lack of contrast between h-cholesterol and the hydrogenous lipids in the rHDL or mature HDL samples. No difference was seen as shown in Figures 4C,D (dotted and dashed lines), which suggests that the phospholipid molecules were primarily exchanged rather than the cholesterol molecules.

Mirroring the NR data for protein incubation alone on model membranes (Figure 2), the model membranes exposed to ApoE4-rHDL required greater structural and compositional changes to obtain a satisfactory fit to the experimental data. Membranes exposed to ApoE3-rHDL were fitted with head and tail region thicknesses kept constant, whereas ApoE3-rHDL on dDMPC + d-cholesterol and all membranes exposed to ApoE4-rHDL required changes to the head and tail thicknesses to find suitable fits. All variations were tested: from keeping them all the same, to only altering the head thickness or to varying all regions. It was found that better fits were possible when allowing greater variation when incubated with ApoE4-rHDL, again indicating greater level of interaction occurring in the presence of ApoE4 compared to ApoE3. This could in part be due to slight embedding of ApoE4 into the hydrophobic core of the membrane, although the resulting membranes were found to maintain, within error, a constant area per molecule across heads and tails. This suggests that the lipid bilayer structure was retained in all cases, however it cannot be disregarded that protein insertion could be balanced in the lipid heads and tails in such a manner that the SLB structure holds. Finally, similar levels of rHDL particles on top of the model membranes were found regardless of ApoE isoform or bilayer composition (approximately 1%).

## Discussion

While the structural difference between the ApoE isoforms is subtle (Chou et al., 2005), clear differences can be seen in their behaviour upon interaction with lipid bilayers. In particular, ApoE4 binds to a larger extent to the membrane core than ApoE3 (Figure 2C), although about the same amount of protein sits on top of the membrane regardless of the lipid type, for each isoform, respectively (Figure 2D). These changes impact the ability of ApoE to bind lipids. In particular, ApoE4 has a greater ability to bind lipids due to structural differences in the helical segments of the C-terminal. This difference leads to a reduced ability to form tetramers, which are less capable than the monomer of binding lipids (Garai et al., 2011; Chetty et al., 2017). ApoE4's increased affinity for lipid binding is also suggested by the fact that ApoE4 preferentially binds to VLDL (for which its surface is about 60% lipids) rather than HDL, whereas ApoE3 binds preferentially to HDL (for which its surface is about 80% protein). Furthermore, ApoE4 lacks flexibility in its C-terminal domain leading to a preference for the lower curvature of the VLDL particles (Hatters et al., 2005; Nguyen et al., 2010). The increased affinity for VLDL leads to an increased clearance of VLDL from plasma *via* ApoE receptors in the liver. However, this in turn leads to LDL receptors being downregulated, raising overall levels of plasma LDL and leading to an increased risk for atherosclerosis (Dong and Weisgraber, 1996; Mahley, 2016a).

Here, we observed that most protein incorporation into bilayers (Figure 2A) occurred within the first hour for the unsaturated lipids but there was further increase with time for saturated ones. Earlier reports on the rate of lipid binding and vesicle solubilisation showed a biexponential decay when solubilising DMPC vesicles at their melting temperature of 24°C (Segall et al., 2002), with ApoE4 presenting a higher rate constant than ApoE3. Another study showed that ApoE4 displays an increased ability to disrupt DMPC vesicles compared to ApoE3 by measuring the release of fluorescent dye (Ji et al., 2002).

Recently, our group showed that native HDL, through its apolipoproteins, present a lower ability to exchange and replace unsaturated lipids as compared to saturated ones (Waldie et al., 2020). Both ApoE variants mirror this trend since these proteins remove more saturated than unsaturated lipids. Atherogenic and amyloid plaques are rich in saturated lipids and cholesterol (Touboul and Gaudin, 2014; Kiskis et al., 2015). In a recent study, a link between AD and the disruption of the metabolism of unsaturated fats such as omega 3 was found, which could provide an explanation for ApoE4's higher affinity for the unsaturated membrane (Snowden et al., 2017). Indeed, a previous report states that supplementing mono- and poly-unsaturated fats in the diet reduce the risk to AD, whereas the introduction of saturated fats increase this risk (Morris, 2004).

The fact that a larger fraction of ApoE4 binds the lipid core compared to ApoE3 and that it displays less sensitivity to the lipid type in the membrane (Figures 2C,D) could be linked to a decreased protein flexibility although NR cannot distinguish whether different ApoE molecules bind to the core and the head or if a single protein bends over across tails and heads. Indeed, ApoE4's greater rigidity forces it to maintain a more compact conformation around the lipid core which, in turn, could render it less able to bind to HDL. Earlier, ApoE4 was shown to disrupt the lipid membrane to a larger extent than ApoE3 (Mahley et al., 2009), in agreement with our results that show larger ApoE4 incorporation into the bilayers and greater structural changes in the SLBs required to fit the NR data after protein incubation. Indeed, ApoE4 could retain a more compact structure folded back on itself due to the Arg-61:Glu-255 salt bridge that could restrain the protein's structure even when associated with lipids (Raussens et al., 1998; Drury and Narayanaswami, 2005; Hatters et al., 2009).

Regarding the rHDL structure, the reports on ApoE-containing particles mainly involve electron microscopy and spectroscopic studies for structural determination (Narayanaswami et al., 2004; Newhouse et al., 2005); and ultracentrifugation (Raussens et al., 1998) and native gel electrophoresis (Raussens et al., 1998; Narayanaswami et al., 2004) for size determination. These reports are in agreement with the size of particles reported here: 10–15 nm in diameter (Figure 3 and Table 2). Contrary to ApoE-rHDL, ApoA1- or membrane scaffold protein (MSP)-based nanodiscs form particles that are about 8–12 nm in diameter (Denisov et al., 2004; Bayburt and Sligar, 2010; Skar-Gislinge et al., 2010; Del Giudice et al., 2017a). A variety of models have been presented to describe protein-lipid particles from the picket-fence model (Jonas et al., 1989), to the double superhelix (Wu et al., 2009) or double belt-like structure (Segrest et al., 1999; Midtgaard et al., 2015), though some of these have faced opposition within the nanodisc community (Jones et al., 2010; Gogonea, 2016). Ellipticity is the main structural feature which differed between the ApoE rHDL, with ApoE4 forming more elongated discs. Our data agree well with the double belt structure since the data suggest that there are two proteins present per nanodisc, also in agreement with ApoA1-rHDL. Previously reported data on ApoE-rHDL show the presence of two or more proteins per disc (Raussens et al., 1998; Narayanaswami et al., 2004; Yamamoto et al., 2008). There has been some debate as to whether ApoE3 and ApoE4 form nanodiscs in a different manner giving structural differences to the protein conformation (de Chaves and Narayanaswami, 2008) and, in turn, their lipid exchange ability. The hypotheses suggested include ApoE3 forming more of an extended belt conformation around the whole perimeter of the disc due to increased flexibility (Narayanaswami et al., 2004). The more compact structure of ApoE4 could lead to potentially more ApoE4 molecules per nanodisc compared to ApoE3 (Gong et al., 2002). However, the same number of molecules were found to be present in both nanodisc variants presented here. The NR lipid exchange data (Figure 4), on the other hand, suggest that the proteins adapt slightly different conformations giving rise to their differing exchange capabilities.

Where some reports have shown an incremental size increase of nanodiscs with increasing protein length (Denisov et al., 2004, 2005), it is of no surprise that the ApoE-rHDL particles reported here are of a slightly larger size than previously reported for ApoA1-rHDL, as the ApoE protein is longer. In turn, the number of lipid molecules per nanodisc is also higher for ApoE- than ApoA1-containing nanodiscs which does not seem to have an effect on the ability of these nanodiscs to exchange lipids: the similar size of ApoE3- and ApoE4-rHDL does not translate into similar functional capabilities, as clear differences were observed in their lipid exchange and removal affinity (Figures 4A,B). Moreover, the lipid exchange (Figure 4B) was similar between ApoE3- and ApoA1-rHDL while it was different between ApoE isoforms, despite them being of similar size but larger than ApoA1 (since the nanodisc concentration was constant across samples, smaller discs imply lower lipid concentration in the case of ApoA1). Therefore, it is clear that the rHDL size (area of the lipid nanodisc) does not determine the extent of lipid exchange; rather, specific apolipoprotein-lipid interactions must be behind the observed phenomena.

ApoA1 and ApoE have similar structures comprising amphipathic alpha-helices that enable them to form nascent HDL-like structures by solubilising phospholipids (Lund-Katz and Phillips, 2010). However, neither ApoE3 nor ApoE4 remove further lipids from the membrane than those exchanged, to a significant degree. This contrasts from what is observed for ApoA1-rHDL, which instead remove a notable quantity of lipids, although to a lesser extent than mature HDL (Figure 4A). The results obtained for ApoA1 are not surprising since it is the more abundant serum HDL protein, whose function is to clear lipids. Our results suggest that the function of ApoE cannot be mainly related to the lipid exchange, but rather to binding ApoE-receptors in the liver. Indeed, the fact that the structure of ApoE4 is more compact than ApoE3 might affect how it binds to ApoE- and other LDL receptors in the liver (Ruiz et al., 2005). However, ApoE-HDL serve as the primary brain lipoproteins, being produced there and lacking the ability to cross the blood-brain barrier (Pitas et al., 1987). The ApoE in the brain is the second most abundant place for ApoE production in the body after the liver (Mahley, 2016a). ApoE plays an important role in maintaining the homeostasis of cholesterol concentration in the brain, where this lipid comprises about 20–25% of the body's total cholesterol (Michikawa, 2006). Whilst HDL in the rest of the body participate in cholesterol efflux thereby removing cholesterol from artery walls, ApoE-HDL in the brain maintain constant cholesterol levels (Mahley, 2016b). Indeed, the current data clearly show that lipid exchange was not affected by the level of deuteration in cholesterol (Figure 4), suggesting that ApoE does not interact with cholesterol significantly. Instead, these results suggest that ApoE-enriched HDL rather transport saturated lipids over cholesterol and unsaturated lipids. By regulating the ratio of saturated lipids in cellular membranes, ApoE-HDL may help maintain membrane elasticity, which is key for the healthy function of the cell. Interestingly, cholesterol crystal nucleation starts sooner in unsaturated than saturated model biles (Halpern et al., 1993), which suggests a higher free cholesterol concentration is achieved in membranes comprised of saturated lipids. Thus, ApoE4 can potentially favour an increase in membrane saturation with cholesterol by exchanging and removing more saturated fats than ApoE3, which could potentially lead to cholesterol crystallisation in the brain.

Even though ApoE4 has a higher affinity to bind lipids than ApoE3 as demonstrated here and elsewhere (Saito et al., 2003), the ability of ApoE isoforms to catalyse the efflux of cholesterol has been disputed: some studies conclude the process is not ApoE isoform dependent (Krimbou et al., 2004) while others have found that ApoE4 has a lesser ability to perform cholesterol efflux compared to ApoE3, especially in relation to neurons in the brain (Huang et al., 1995; Gong et al., 2002; Michikawa et al., 2007). This lack of ability of ApoE4 to remove and deposit cholesterol in the brain efficiently has been proposed to be one of the main reasons for the onset of AD (Gong et al., 2002; de Chaves and Narayanaswami, 2008). Our simplified model shows that neither ApoE isoform has a special affinity for taking up cholesterol and it is therefore hypothesised that there is no significant difference in their capacity to efflux cholesterol.

## Conclusions

Neutron reflection results suggest that ApoE4 adopts a different conformation to ApoE3 at model membranes, and that this conformation differs between saturated and unsaturated membranes for ApoE3 only. Moreover, neither ApoE isoform removes a significant amount of unsaturated lipids from the model membrane used but they were able to remove saturated lipids to similar extents. Small-angle neutron scattering was used to demonstrate that the structure of nascent-like rHDL particles made with DMPC and either ApoE3 or ApoE4 is similar, forming elliptical disc-like structures. Neutron reflection was then used to quantify the extent of lipid exchange and lipid removal by nascent-like rHDL particles and model membranes. The data show that either ApoE3- or ApoE4-rHDL particles have a low ability to remove saturated lipids as compared to ApoA1-rHDL or mature HDL. The extent of lipid exchange, on the other hand, is similar between the isoforms and is impaired by the presence of cholesterol. Finally, ApoE does not exchange or remove cholesterol molecules but rather saturated lipids. The results here mirror the physiological roles of ApoE-HDL and ApoA1-HDL particles in the brain and in serum respectively. This demonstrates that our models are suitable to study the function of these particles in a range of experimental conditions.

## Data Availability Statement

The datasets presented in this study can be found in online repositories. The names of the repository/repositories and accession number(s) can be found at: DOIs: 10.5291/ILL-DATA.9-13-807 (FIGARO, June, September 2019), 10.5291/ILL-DATA.9-13-894 (FIGARO, February 2020) and 10.5291/ILL-DATA.8-03-979 (D11, September 2020).

## Author Contributions

SW produced the ApoE and ApoE-rHDL, participated in the planning of the experiments, performed all experiments, analysed all of the NR and SANS data and wrote the initial draft of this article. FS, NP, YG, SP, and MC participated in performing some of the experiments. FS, FR-R, and MC supported the data analysis. SW and MM produced the deuterated cholesterol and HP purified it. RDG produced the ApoA1-rHDL. TD and NY produced the POPC used. MM, SM, VF, MH, and MC participated in the planning of the experiments. MM, MH, and VF supervised activities in the ILL's Life Sciences Group. MC obtained the funding and supervised the work. All authors contributed to the discussions and editing of the manuscript.

## Conflict of Interest

The authors declare that the research was conducted in the absence of any commercial or financial relationships that could be construed as a potential conflict of interest. The reviewer DH declared a past co-authorship with one of the authors VF.

## Footnotes

^1^http://www.sasview.org/.
