# Supplementary material for: ApoE and ApoE Nascent-Like HDL Particles at Model Cellular Membranes: Effect of Protein Isoform and Membrane Composition
Source: Front Chem. 2021 Apr 29;9:630152. doi: 10.3389/fchem.2021.630152 (PMC8117676; doi:10.3389/fchem.2021.630152)
Supplement: Supplementary file 1 [file Data_Sheet_1.PDF]

Supplementary Material

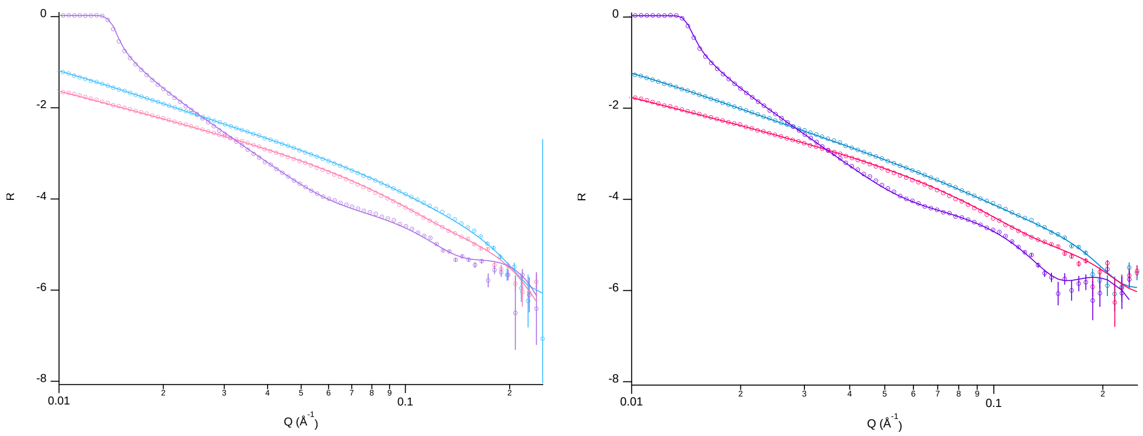

Figure S1. NR data and best fits of dDMPC model membrane before (left) and after (right) incubation with ApoE3 protein. dTBS, cmSi and hTBS contrasts are shown in purple, pink and blue respectively.

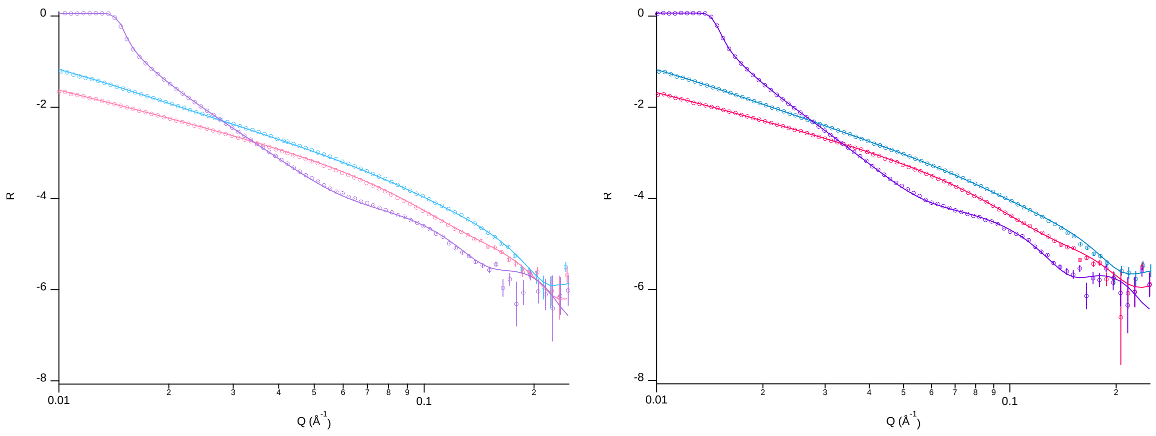

Figure S2. NR data and best fits for dPOPC model membrane before (left) and after (right) incubation with ApoE4 protein. dTBS, cmSi and hTBS contrasts are shown in purple, pink and blue respectively.

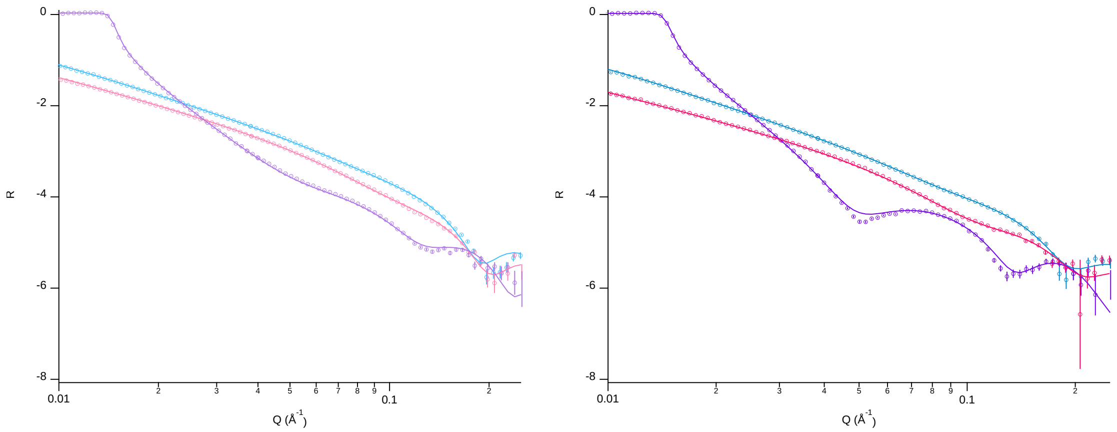

Figure S3. NR data and best fits for dDMPC and d-Cholesterol model membranes before (left) and after (right) incubation with ApoE3 based rHDL. dTBS, cmSi and hTBS contrasts are shown in purple, pink and blue respectively.

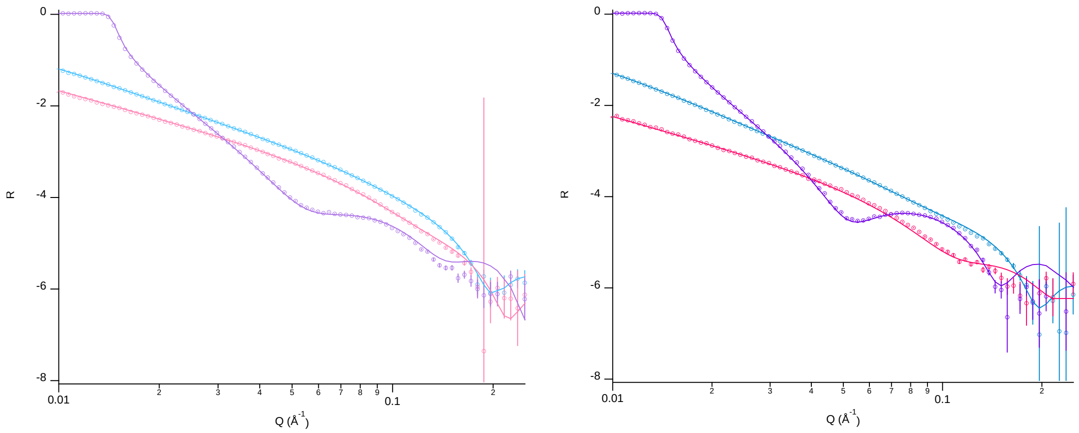

Figure S4. NR data and best fits for dDMPC and h-cholesterol model membranes before (left) and after (right) incubation with ApoE3 based rHDL. dTBS, cmSi and hTBS contrasts are shown in purple, pink and blue respectively.

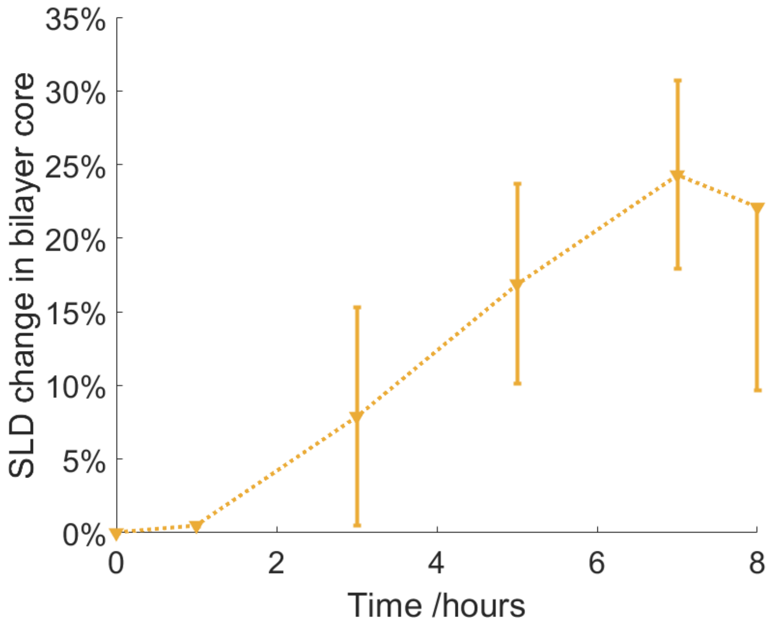

Figure S5. Kinetics of incubation of ApoA1-rHDL with dDMPC and d-cholesterol model membranes at 37 °C in h-Tris buffer. The errors are calculated from the two possible extremes from full lipid removal to full lipid exchange, since there is only one contrast it is not possible to independently distinguish the extent of either lipid removal or lipid exchange.

Table S1. Best fit parameters for pristine model membranes before incubation with either lipid-free or rHDL-bound ApoE. The parentheses detail which sample was incubated on that SLB. * Indicates fixed value. ** Indicates calculated values.

**Bilayer**	**Layer**	**Thickness (Å)** E± 0.1-1	**SLD (*10^{-6} Å^{-2})** E± 0.01-0.3	**Solvent (%)** E± 0.2-5	**MMA** (Å^2)** E±1-5
**dDMPC (E3 prot)**	Inner Head	8.98	1.89*	35	56
	Tail	25.2	6.46	4.8	65
	Outer Head	8.98	1.89*	35	56
**dPOPC (E3 prot)**	Inner Head	7.8	1.89*	31	60
	Tail	28.4	6.45	16	78
	Outer Head	7.8	1.89*	31	60
**dDMPC (E4 prot)**	Inner Head	8.98	1.89*	37.7	58.2
	Tail	27.2	6.45	3.5	59.6
	Outer Head	8.98	1.89*	37.7	58.2
**dPOPC (E4 prot)**	Inner Head	8.8	1.89*	46.4	70
	Tail	28.6	6.25	14	76
	Outer Head	8.8	1.89*	46.4	70
**dDMPC (E3 disc)**	Inner Head	9.46	1.89*	38	55.6
	Tail	26.2	6.34	4.4	62.4
	Outer Head	9.46	1.89*	38	55.6
**dDMPC-hcholesterol (E3 disc)**	Inner Head	7.7	1.89*	15	50
	Tail	30.7	5.7	6	53
	Outer Head	7.7	1.89*	15	50

**dDMPC-dcholesterol (E3 disc)**	Inner Head<br>Tail<br>Outer Head	8.5<br>32.1<br>8.5	1.89*<br>6.58<br>1.89*	29<br>2<br>29	54<br>48.5<br>54
**dDMPC (E4 disc)**	Inner Head<br>Tail<br>Outer Head	9.4<br>26<br>9.4	1.89*<br>6.22<br>1.89*	33<br>0.5<br>33	52<br>61<br>52
**dDMPC-hcholesterol (E4 disc)**	Inner Head<br>Tail<br>Outer Head	9.45<br>31.3<br>9.45	1.89*<br>5.7<br>1.89*	26<br>5.8<br>26	46.6<br>51.7<br>46.6
**dDMPC-dcholesterol (E4 disc)**	Inner Head<br>Tail<br>Outer Head	8.9<br>26.3<br>8.9	1.89*<br>6.43<br>1.89*	26<br>3.2<br>26	49.5<br>59.9<br>49.5
**dDMPC-dcholesterol (A1 disc)**	Inner Head<br>Tail<br>Outer Head	6.9<br>33.6<br>6.9	1.89*<br>6.59<br>1.89*	19<br>5.4<br>19	58<br>48<br>58

Table S2. Best fit parameters for model membranes after incubation with either lipid-free or rHDL-bound ApoE. * Indicates fixed value. ** Indicates calculated values.

**Bilayer**	**Layer**	**Thickness (Å)** E± 0.1-1	**SLD (*10^{-6} Å^{-2})** E± 0.01-0.3	**Solvent (%)** E± 0.2-5	**MMA** (Å^2)** E±1-5
**dDMPC E3 prot**	Inner Head	8.98	1.89*	49.2	71
	Tail	25.2	6.08	14.7	73
	Outer Head	8.98	1.89*	44.2	65
	prot	28*	2.99*	94.9	
**dPOPC E3 prot**	Inner Head	7.82	1.89*	35	64
	Tail	28.4	5.75	13	76
	Outer Head	7.82	1.89*	42	71
	prot	28*	2.99*	94.8	
**dDMPC E4 prot**	Inner Head	8.8	1.89*	42	64
	Tail	26.8	5.94	17.7	71
	Outer Head	7.1	1.89*	39	75
	prot	28*	2.99*	96.4	
**dPOPC E4 prot**	Inner Head	8.8	1.89*	47.2	70
	Tail	29.4	5.78	13.5	73
	Outer Head	7.6	1.89*	41	73
	prot	28*	2.99*		
**dDMPC E3 disc**	Inner Head	9.46	1.89*	40.3	57.7
	Tail	26.2	3.65	0.3	59.9
	Outer Head	9.46	1.89*	43	60
	disc	44*	2.56*	98.9	

**dDMPC-hcholesterol E3 disc**	Inner Head	7.7	1.89*	14	49
	Tail	30.7	3.61	3.2	51
	Outer Head	7.7	1.89*	18	52
	disc	44*	2.56*	99	
**dDMPC-dcholesterol E3 disc**	Inner Head	6.7	1.89*	20	61
	Tail	30.9	5.26	5.2	51.2
	Outer Head	9.2	1.89*	25	47
	disc	44*	2.76*	98.7	
**dDMPC (E4 disc)**	Inner Head	7.5	1.89*	40	73
	Tail	32.3	3.86	7.9	53
	Outer Head	6.2	1.89*	30	75
	disc	44*	2.52*	98.9	
**dDMPC-h-cholesterol E4 disc**	Inner Head	10.9	1.89*	36.8	47
	Tail	30.9	3.67	0	49.3
	Outer Head	9.3	1.89*	21	44
	disc	44*	2.52*	98.8	
**dDMPC-dcholesterol E4 disc**	Inner Head	6.6	1.89*	23	64
	Tail	29	4.67	8.6	56.6
	Outer Head	8.2	1.89*	38	64
	disc	44*	2.52*	97.2	
**dDMPC-dcholesterol A1 disc**	Inner Head	6.7	1.89*	35	75
	Tail	34.6	5.12	18.8	54
	Outer Head	9.4	1.89*	31	50
	disc	44*	2.5*	96.8	
